# Sounds and Sights of Motivation: Using Digital Encouragement and Dissociation Strategies during Cardiopulmonary Exercise Testing To Improve Patient Engagement and Diagnostic Quality

**DOI:** 10.1186/s40798-025-00847-4

**Published:** 2025-04-24

**Authors:** Sascha Ketelhut, Ralf Brand, Anna Lisa Martin-Niedecken, Daniel Hug

**Affiliations:** 1 Institute of Sport Science, University of Bern, Bremgartenstrasse 145, Bern, 3013 Switzerland; 2 Sport and Exercise Psychology, University of Potsdam, Potsdam, Germany; 3 Institute for Design Research, Zurich University of the Arts, Zurich, Switzerland; 4 Institute for Computer Music and Sound Technology, Zurich University of the Arts, Zurich, Switzerland

**Keywords:** Encouragement, Cardiopulmonary Exercise Testing, Audio, Video, Dissociation, Dual-mode Theory

## Abstract

The cardiopulmonary exercise test (CPET) stands as a fundamental assessment in sports and health science as it is crucial for evaluating physical fitness, tailoring training regimens, and diagnosing health conditions. An essential aspect of this test is that participants exert maximal effort, as insufficient effort can compromise the validity of the results. While reliable results are seen in physically active individuals, reliability may not be guaranteed in exercise-naïve, less fit, and clinical populations lacking experience to exhaust themselves. This can result in inaccurate assessments, misdiagnoses, misinterpretation of intervention results, and unsuitable exercise recommendations. Various strategies, including verbal, audio, and video stimuli, are used to elicit maximal effort in exercise. While music and verbal encouragement are well-studied, non-musical sound, video, virtual reality, and augmented reality remain underexplored, with inconsistent or absent CPET-specific guidelines. Surprisingly, innovative approaches combining multisensory digital methods are notably lacking. Future research should systematically evaluate these strategies to create more immersive and engaging experiences, increasing effort and standardizing encouragement. Adaptive audio-visual methods could improve test reliability, validity, and workflows while enhancing participant enjoyment. Realizing this potential requires interdisciplinary collaboration among sound, graphic, and video designers, exercise physiologists, and psychologists. By moving beyond conventional approaches, CPET could be transformed into a more engaging and effective tool for diverse populations.

## Key Points

• Cardiopulmonary exercise testing is essential for assessing fitness, monitoring progress, and diagnosing health conditions in sports and clinical settings. However, ensuring participants exert maximal effort is challenging and affects test validity.

• Various strategies like verbal encouragement and audio/video stimuli are used to motivate participants during testing. Yet, a comprehensive understanding and standardized guidelines for these methods in the context of cardiopulmonary exercise testing are lacking, and innovative digital approaches are missing.

• Exploring novel multisensory methods, including adaptive digital audio-visual elements, can create immersive testing experiences, boost motivation, and standardize encouragement. This enhances test reliability and comparability and may foster participants’ compliance with interventions.

## Introduction

Cardiopulmonary exercise testing has significant applications in both sports and clinical settings. Despite the numerous measures implemented to ensure reliable and valid test results, one critical aspect often overlooked in research and practice is the influence of encouragement. This oversight can significantly affect test outcomes, potentially compromising their accuracy and reliability. In this current opinion, we aim to highlight the importance of encouragement and dissociation strategies during cardiopulmonary exercise testing and to stimulate the development of innovative approaches for providing effective encouragement.

Maximal exercise testing serves as a tool for physical fitness assessment, monitoring progress, tailoring training regimens, diagnosing various health conditions, and helping healthcare professionals in the development of personalized treatment and rehabilitation strategies [1–3]. Overall, maximal exercise testing is a valuable diagnostic tool that contributes to improved patient care in clinical practice [2].

Among the numerous tests applied in research and practice, the cardiopulmonary exercise test assessing cardiorespiratory fitness stands out as one of the most significant assessments in the field of sport and health science as accumulated evidence suggests that cardiorespiratory fitness is one of the strongest predictors of all-cause mortality [4, 5]. Cardiorespiratory fitness can be “evaluated through maximal oxygen uptake (VO_2_max), denoting the peak level of oxygen consumption achievable during a rigorous cycling or treadmill test performed until exhaustion [6]. The evaluation of VO_2_max holds significant clinical value in identifying and comprehending issues related to various health conditions affecting the pulmonary, cardiovascular, and muscular systems. Furthermore, it can non-invasively evaluate the effectiveness of exercise training programs and other performance-enhancing approaches in both healthy individuals and those with health conditions [7].

A crucial aspect of cardiopulmonary exercise testing is that it relies on participants exerting their maximal effort. Insufficient effort levels can compromise the validity of test results [7, 8]. In young and healthy individuals accustomed to pushing themselves to exhaustion, the cardiopulmonary exercise test produces a highly reproducible VO_2_max [9]. However, this reliability may not be guaranteed in exercise-naïve, unmotivated, less fit, overweight/obese, older adults, and/or clinical populations who lack experience to exert themselves to exhaustion [7]. This could lead to inaccurate performance assessments, misdiagnoses, and the adoption of unsuitable exercise and treatment strategies. Additionally, when evaluating a training or therapeutic approach, there is a risk of overestimating effectiveness due to repeated testing as participants gain experience with the test [7]. While submaximal tests aim to extrapolate maximal values from physiological parameters during submaximal exercise, direct measurement through maximal testing remains the only accurate and valid method for determining an individual’s true maximal capacity [10].

Various strategies, including verbal, audio, and video stimuli, have been employed in research and practice to encourage maximal effort in exercise testing. The use of music in exercise settings is both widely implemented and grounded in established conceptual frameworks [11–13]; however, the understanding of other strategies, such as non-musical sound, video, virtual reality, and augmented reality, remains relatively limited. Moreover, specific recommendations for cardiopulmonary exercise testing remain either inconsistent or entirely lacking [14]. Surprisingly, innovative approaches leveraging the diverse properties of digital methods and combining various strategies are conspicuously absent.

We urge a departure from long-standing conventional encouragement methods and encourage the exploration and development of novel approaches that improve the overall testing experience, increase the willingness to exert maximal effort and contribute to improving standardization for greater test reliability during cardiopulmonary exercise testing. In particular, we call for investigating digital and multisensory approaches as a particularly promising avenue in this pursuit.

## Affective Responses To Exercise

Previous research indicates that individuals often terminate a cardiopulmonary exercise test well before perceiving any physical limitation [15–17], citing reasons such as perceptions of physical discomfort, pain, safety concerns, and the achievement of self-imposed goals [16]. This is particularly notable among the inactive, children, obese individuals, older adults, and patients at risk [7, 18]. Although exercising at moderate intensity (below the ventilatory threshold) typically increases pleasure for most individuals, there is a decline in pleasure at severe intensities (at and above the ventilatory threshold) [19]. The decline can be attributed to elevated physiological responses, such as increased blood lactate, oxygen consumption, and heart rate, which trigger a generalized stress response. The universal decline in affective valence beyond the ventilatory threshold contributes to the perception of intolerability, making further continuation feel unsustainable [19, 20]. Additionally, evidence suggests that a decreased prefrontal activation combined with increased activation of the amygdala account for the observed decline in affective valence [21].

According to dual mode theory, diverting attention away from bodily sensations, particularly during exercise at the ventilatory threshold, can improve mental states (less unpleasant core affect and more voluntary effort) [22]. Previous research suggests that various methods and strategies for encouraging participants (see the following section) can help distract them from unpleasant physical sensations during exercise, thereby helping them regulate affective responses [23]. Dissociation strategies - such as focusing on external distractions or mental imagery - are most effective at moderate exercise intensities, particularly near the ventilatory threshold [24]. At this level, physiological sensations of exertion and discomfort remain manageable, as dissociative strategies, such as listening to words or music, attract attention to external stimuli, reducing awareness of bodily signals [22]. However, beyond the respiratory compensation point - where a physiological steady state can no longer be maintained - increasing ventilation, and perceived exertion dominate awareness. These intense and overwhelming interoceptive signals make it increasingly difficult to sustain dissociative strategies, as the body’s distress demands attentional focus and overrides attempts at distraction. Research shows that the quality and degree of the dissociation are crucial factors in counteracting the interoceptive afferents generated by increasing exercise intensity [22]. Consequently, the question arises whether more immersive strategies - where the quality, degree, and timing of dissociation are optimized - can yield similarly beneficial effects on mental states or subjective experiences, even beyond the respiratory compensation point.

### Strategies for Encouragement and Dissociation

Verbal encouragement is often recommended in exercise testing guidelines [25] as a strategy to increase motivation during exercise testing. Empirical evidence has demonstrated that verbal encouragement leads to noticeably higher effort investment during exercise testing [26, 27]. Studies indicate significant increases in time to exhaustion, maximal heart rates, VO_2_max, and blood lactate levels in various maximal exercise tests [8, 26, 28]. Furthermore, improvements in affect [29], alterations in motor unit activation [30], as well as enhancements in movement efficiency and effectiveness, have been reported [31].

Apart from verbal encouragement, music or non-musical sound can also promote an ergogenic effect by either reducing perceptions of fatigue or increasing work capacity [32, 33]. Research shows that music positively influences core affect and mood, enhances self-efficacy, and can help in dissociating from exertion-induced unpleasant sensations during exercise [34–36]. For instance, listening to music before or during an exercise test has been linked to positive effects on performance [37]. Hutchinson et al. [38] reported that participants experienced an increase in power output when listening to music during a supramaximal exercise bout, along with higher reported levels of task motivation and positive affect. In a study conducted by Elliott et al. [39] untrained participants covered a greater distance in a submaximal cycling task and reported higher levels of enjoyment when listening to music. However, the benefits of music appear to be most consistently evident when engaging in low-to-moderate intensity exercise [32, 40]. At higher intensity levels (85% of maximal heart rate), music has been shown to lose its beneficial effect [41]. This aligns with dual mode theory postulates (see the previous section) as well as the the parallel-processing model, indicating a threshold of exercise intensity where focus naturally shifts to associative processing, rendering external cues less effective for maintaining a dissociative focus [42]. However, well-selected music appears to promote positive affect even at very high exercise intensities [36, 43]; nonetheless, the literature remains inconclusive [44].

Currently, the majority of studies in this field focus primarily on precomposed music pieces. However, sonification - the process of transforming data into sound, which can include tonal elements or various types of noise - has emerged as a promising alternative [45]. Increasing evidence suggests that sonification and non-musical auditory displays (e.g., sound effects) can enhance movement precision, optimize movement efficiency, increase muscular activation, improve performance, and support motor control [33, 46, 47]. Notably, performance improvements have also been demonstrated during maximal exercise tests [45].

Researchers propose that video-based content or visual media can be effective during exercise to achieve a dissociative focus, enhancing adherence [48] and even improving performance during an all-out exercise task [49]. The potential of video to attain a dissociative focus is underscored by the fact that vision tends to have a more significant impact on perceptions, judgments, and overall understanding than other sensory inputs [50]. Unfortunately, research on the ergogenic effect of video is limited, but existing studies indicate that displaying videos during vigorous exercise (75–85% maximal oxygen consumption) can reduce perceived exertion and enhance exercise economy [51, 52]. In a recent study [53], patients who watched a virtual walking group during the 6-minute walking test exhibited significantly higher exercise capacity, a longer time to exhaustion, greater comfort, and a higher self-rated closeness to physical exertion compared to those who viewed a static image. However, Chow et al. [42] found that participants exercising at 125% of the ventilatory threshold could not maintain a dissociative attention focus when presented with video only. These results are supported by Barwood et al. [54] showing that music or video alone did not influence attentional focus or perceived exertion during a 15 min maximal effort run. However, the combination of video and audio has been shown to result in a higher degree of dissociation, reduced perception of exertion, more positive affective responses, and increased state of motivation than either modality alone [22, 23, 42]. This effect is commonly interpreted as being the result of the combined audio and video engaging two senses (hearing and sight), thus mounting a more powerful sensory competition against exercise-induced interoceptive afferents [22].

## Reconsidering Strategies for Encouragement and Dissociation

As the cardiopulmonary exercise test stands as a fundamental assessment in sports and health science, considerable efforts are invested in standardizing test conditions and enhancing result reliability. However, a noticeable disparity in the implementation of encouragement strategies exists, highlighting a substantial gap in achieving standardized encouragement. This gap not only manifests among different test personnel conducting the test but also within the same individual across multiple test instances. Addressing and minimizing this divergence in encouragement approaches during testing is paramount to enhancing the overall reliability and validity of cardiopulmonary exercise testing in both sports science and clinical settings.

The most common strategy is, by far, verbal encouragement provided by the test personnel. Despite ongoing investigations into the attributes of effective verbal encouragement, a lack of established guidelines persists [14]. The implementation of recommendations for verbal encouragement, encompassing content, timing, tone, and frequency, faces significant challenges in achieving high standardization in everyday practice. Even if guidelines existed, it is questionable whether they could be adequately implemented. While the standardization of content and timing may seem straightforward, challenges arise in standardizing elements such as tone, pitch, and volume. Addressing this concern, the integration of digitized verbal encouragement holds promise for enhancing standardization. Given the significant research gap, there is an urgent need for comprehensive studies in this domain.

Building upon these insights, there is potential to develop specially designed programs and tools capable of delivering verbal encouragement at optimal times with appropriate sonic properties, and content. Leveraging advanced algorithms and artificial intelligence, real-time data analysis can be employed to dynamically adapt encouragement strategies based on the user’s physiological responses (e.g. heart rate, respiratory exchange ratio, etc.), thereby ensuring adaptive and personalized encouragement. However, while striving for personalized encouragement, it is crucial not to compromise standardization. Therefore, a consistent level of encouragement within each individual must be guaranteed. The goal is to ensure that every person is motivated to perform at their maximum capacity, thereby enhancing the reliability of test outcomes.

While it may initially appear unconventional to certain practitioners, the fitness sector’s success in replacing traditional coaching methods with digital alternatives serves as compelling evidence for the viability of technological interventions in motivating individuals during exercise. Moreover, the COVID-19 pandemic has notably accelerated the acceptance and seamless integration of digital coaching solutions into mainstream practices [55].

In addition to verbal encouragement, music is frequently used to motivate individuals during exercise, shift their attentional focus towards dissociation, and improve affective responses. Apart from music, sonification and non-musical auditory displays are further auditory options that have been shown to affect movement execution and improve exercise performance [45, 47].

Even though the ergogenic effects of music, sonification, or auditory displays are promising [36, 43], the utilization of audio encouragement strategies during cardiopulmonary exercise testing remains sparse. Predominantly, pre-existing music or basic auditory displays (e.g. beeps) are employed during exercise testing. However, the suitability of these stimuli in meeting the recommendations for effective audio encouragement [32] is questionable. To leverage the full potential of audio encouragement it is imperative to design stimuli that conform to current research recommendations and are tailored for the specific test procedure, target group, and level of exhaustion. Moreover, a compelling strategy to increase effort investment during exercise testing may be the seamless integration of verbal encouragement into these auditory displays.

While the utilization of video, particularly when combined with audio, shows promise as an effective encouragement strategy [22, 23, 42], the current body of research lacks explicit recommendations for designing videos to establish a truly motivational climate. Notably, many studies have relied on asynchronized video files not directly aligned with the exercise (e.g. speed, content). To unlock the complete potential of video as an encouragement tool, there is a compelling need to delve into creating adaptive video stimuli intricately linked to the exercise task, allowing for real-time adjustments in speed and content. Furthermore, incorporating storytelling into the video stimulus by weaving a narrative around the exercise task can help elicit emotions, capture attention, and sustain participant engagement [56–58]. Narration can further help contextualize the exercise task by offering insights into the test environment, elucidating the purpose of the activity, or the benefits of exerting effort.

Aside from the content, the presentation of video stimuli plays a crucial role in enhancing interactive and immersive experiences [59]. In this regard, virtual reality (VR) and augmented reality may be promising approaches that can transport participants into realistic and engaging virtual environments, effectively occluding them from physical reality and accommodating a range of senses [60, 61]. One of the key advantages of VR is its ability to create a sense of presence, wherein individuals feel as though they are physically present within the virtual environment. This can make participants feel more connected to the exercise task [62] and has been shown to improve affective, and perceptual responses [63, 64]. Given the widespread accessibility of VR technologies, the integration of VR-supported video stimuli into cardiopulmonary exercise testing warrants consideration.

The integration of digital encouragement strategies into cardiopulmonary exercise testing presents significant opportunities for enhancing patients’ effort investment. However, it is crucial to recognize and address potential risks associated with these approaches. One major concern is the possibility of participants becoming overly reliant on digital stimuli, which could lead to a disconnect from their own bodily signals (i.e., weakened interoception). Excessive reliance on auditory and visual cues might hinder individuals’ ability to accurately perceive physical limitations or important symptoms (e.g. severe dyspnea, angina pectoris symptoms) during exercise. While physical parameters can be closely monitored and the risk of adverse events minimized by considering termination criteria, future research should explore potential negative side effects. Another risk is the development of dependency on digital encouragement strategies over time. If participants rely too heavily on external stimuli for motivation during exercise testing, they may struggle to maintain motivation or exert effort without these stimuli. This could undermine the effectiveness of exercise interventions or rehabilitation programs in the long term, as participants may not develop intrinsic motivation or self-regulation skills necessary for sustained behavior change beyond the testing environment. Therefore, while digital approaches offer exciting possibilities for improving motivation and engagement during exercise testing, thorough research is essential to understand and mitigate any potential negative effects on participant performance and test validity.

## Future Directions

In the context of the cardiopulmonary exercise test, where critical information is derived, the imperative to standardize encouragement becomes evident. Future research efforts should focus on systematically exploring the effectiveness of various digital encouragement strategies, including verbal, audio, and video stimuli synchronized with test protocols. Currently, to the best of our knowledge, studies comparing different encouragement strategies during cardiopulmonary exercise testing are lacking. Therefore, within-subject crossover studies are warranted to evaluate the effectiveness of various strategies while systematically varying key characteristics (e.g., timing, intensity, and type) of each approach to ensure robust and individualized findings. However, a critical challenge is that testing too many strategies within the same individual may be problematic due to the strong familiarization effects associated with the cardiopulmonary exercise test [65].

Integrating encouragement strategies into programs adaptable to various test protocols and equipment has the potential to revolutionize the way to provide encouragement during cardiopulmonary exercise testing. Insights gained from digital innovations in the fitness sector, as demonstrated by platforms like Les Mills, Nordictrack, and Zwift, underscore the potential that multisensory digital approaches could have during performance diagnostics.

The digitalization of encouragement strategies not only has the potential to address biases associated with non-standardized practices, thereby improving test reliability and validity. The incorporation of adaptive digital audio-visual encouragement can cultivate a more immersive, engaging, and enjoyable testing experience. This may even have the potential to positively shape participants’ attitudes toward physical activity and exercise [66–68] leading to increased long-term engagement in physical activities and improved compliance with interventions. Furthermore, the integration of digital tools could contribute to streamlining workflows during testing procedures, enabling test personnel to concentrate more on the patient and monitoring and interpreting the test data.

A significant challenge in encouragement lies in the personalized nature of the experience. Participants may respond differently to various encouragement strategies. What motivates one person might not be effective for another, making it challenging to identify universally successful approaches. In this regard, exploring digital approaches capable of identifying personality types through integrated assessments and adjusting encouragement accordingly would be intriguing. Regarding the development process, it is imperative to employ user-centered design processes to conceive adequate encouragement strategies for specific target groups and personality types.

## Conclusion

In summary, we advocate for a paradigm shift in the application of encouragement strategies during cardiopulmonary exercise tests, urging the adoption of digital approaches to optimize reliability, validity, and workflows. In this context, an interdisciplinary approach is fundamental, particularly emphasizing the critical contributions of exercise physiologists and psychologists, along with the insights and skills of sound designers and graphic/video designers. The transformative impact of such digital innovations holds the potential to significantly advance sports science and clinical assessments, marking a new era in exercise diagnostics.

## Data Availability

Not applicable.
